# Incidence of post-traumatic hydrocephalus in traumatic brain injury patients that underwent DC versus those that were managed without DC: A systematic review and meta-analysis

**DOI:** 10.1016/j.bas.2021.100303

**Published:** 2021-10-22

**Authors:** Georgios Mavrovounis, Adamantios Kalogeras, Alexandros Brotis, Corrado Iaccarino, Andreas K. Demetriades, Konstantinos N. Fountas

**Affiliations:** a Department of Neurosurgery, Faculty of Medicine, University of Thessaly, Larisa, Greece; b Division of Neurosurgery, Department of Biomedical, Metabolic and Neural Sciences, University Hospital of Modena, University of Modena and Reggio Emilia, Modena, Italy; c Department of Neurosurgery, New Royal Infirmary, Edinburgh, UK

**Keywords:** Traumatic brain injury, Post-traumatic hydrocephalus, Decompressive craniectomy, Intracranial pressure, TBI, Traumatic Brain Injury, GCS, Glasgow Coma Scale, PTH, Post-traumatic hydrocephalus, CSF, Cerebrospinal fluid, DC, Decompressive Craniectomy, ICP, Intracranial pressure, RCT, Randomized Controlled Trial, RoB 2.0, Risk of Bias 2.0, OR, Odds Ratio, CI, Confidence Interval

## Abstract

**Introduction:**

There is an ongoing debate whether Decompressive Craniectomy (DC) serves as an independent risk factor for the development of Post-traumatic Hydrocephalus (PTH).

**Research question:**

The aim of this systematic review and meta-analysis was to compare the incidence of PTH in TBI patients that underwent DC versus those that were managed without DC.

**Materials and methods:**

The literature was systematically reviewed to identify studies with specific inclusion criteria: (1) Randomized Controlled Trials and observational studies with more than 10 patients in each study arm, (2) comparing the incidence of PTH, (3) in patients aged ≥15 years old, (4) that either underwent DC or received other treatment (non-DC). (5) Only studies in English were included and (6) no restrictions were applied on publication date. The pooled Odds Ratio (OR) and Confidence Interval (CI) were calculated. The quality of the included studies was assessed using the ROBINS and RoB 2.0 tools.

**Results:**

Evidence from six articles was synthesized, incorporating data from 2522 patients. A statistically significant higher occurrence of PTH [OR (95% CI): 4.84 (2.51, 9.31); Pz ​< ​0.00001] was identified in patients undergoing DC for TBI when compared to those that were managed without DC. The same was true when only patients with severe TBI were included in the analysis [OR (95% CI): 2.87 (1.85, 4.43); Pz ​< ​0.00001].

**Discussion and conclusion:**

Our study has shown, within limitations, a clear association between DC and PTH. Further prospective studies, providing high-quality evidence, are needed to definitively establish any causative relationship between DC and PTH.

## Highlights

• TBIs are associated with the development of PTH.
• Patients undergoing decompressive craniectomy are more likely to develop PTH.
• Further studies should be conducted to establish a causative relationship.

## 1 Introduction

Traumatic brain injury (TBI) constitutes a major cause of morbidity and mortality amongst the general population, and presents an important medico-social issue worldwide, with severe financial burden (Maas et al., 2017). The physical and mental health of patients that survive the initial traumatic event is often severely affected, as they suffer from long-term disabilities (Ma et al., 2014). This burden is often amplified by the inconsistency of healthcare access at a local, national and international level, and the weak evidence pertaining the medical, surgical, and rehabilitation management of TBI patients (Maas et al., 2017).

One of the most well-recognized sequelae of TBI, especially in patients with severe brain trauma (Glasgow Coma Scale; GCS <9), is the development of post-traumatic hydrocephalus (PTH). The initial traumatic event as well as the physiological and anatomical changes that occur after the injury can alter the Cerebrospinal fluid (CSF) hydrodynamics (Guyot and Michael, 2000). PTH can develop weeks to months after the initial brain injury and its incidence varies widely in the literature, mainly due to the heterogeneity of the available definitions and the implemented clinical and/or imaging criteria (Fotakopoulos et al., 2016). As mentioned by the Lancet Neurology Commission, “trauma disturbs the brain in complex ways, affecting multiple outcome domains” (Maas et al., 2017).

Decompressive craniectomy (DC) is a commonly implemented treatment strategy in patients with TBI, especially when intractable intracranial hypertension has developed (Cooper et al., 2011; Hutchinson et al., 2016). It is effective in lowering the intracranial pressure (ICP) (Hutchinson et al., 2016); however, despite reduction of mortality, a higher incidence of unfavorable outcomes, including PTH (Ding et al., 2014), when compared with conservative, non-surgical treatment (Cooper et al., 2011) has been associated with DC. Currently the direct link between DC and the development of PTH is not confirmed in all the available studies in the literature and hence remains controversial.

The aim of the current systematic review and meta-analysis was to compare the incidence of PTH in patients undergoing DC versus those who were managed without DC.

## 2 Methods

The protocol for the current systematic review was formulated and written according to the PRISMA checklist (Moher et al., 2009), and was registered on the International Prospective Register of Systematic Reviews (PROSPERO ID: CRD42021224759); it is available in full at: https://www.crd.york.ac.uk/prospero/display_record.php?RecordID=224759.

### 2.1 Literature search

An electronic search of the PubMed (MEDLINE), Scopus, and clinicaltrials.gov databases was performed by two of the authors (M.G., K.A.), independently. The search algorithms used contained the following words combined with the Boolean operators “AND” and “OR”, as appropriate: craniotomy, craniectomies, craniectomy, decompressive craniectomy, craniocerebral trauma, traumatic brain injuries, closed head injuries, brain injuries, traumatic head, traumatic brain, head trauma, brain trauma, head injury, hydrocephalus, post-traumatic hydrocephalus, posttraumatic hydrocephalus, post traumatic hydrocephalus. The exact search algorithm for each database is presented in Appendix B. To identify additional studies, the reference lists of the retrieved articles were manually reviewed. The last literature search was performed on January 28th, 2021.

### 2.2 Inclusion and exclusion criteria

We included (1) randomized controlled trials (RCTs) and observational studies with more than 10 patients in each study arm, (2) comparing the incidence of PTH, (3) in patients aged 15 years old or older, (4) with TBI, (5) who either underwent DC or received other treatment (non-DC). (6) Only studies with available full texts written in English were included, while (7) no restrictions were applied on publication date. Furthermore, we excluded (1) in vitro and animal studies, underpowered observational studies, case reports, editorials, abstracts, and white papers. We also excluded studies (2) focusing on pediatric patients, (3) on patients with other intracranial pathologies (e.g. spontaneous intracranial hemorrhage, ischemic stroke, Chiari malformation), and (4) without quantitative data pertinent to our analysis.

### 2.3 Data extraction

Two of the authors (M.G., K.A.) used an Excel form to independently perform the data extraction. The following data were extracted: first author’s name, year of publication, country of origin of the patients included in the studies, enrollment period, mean/median age, male/female ratio, injury severity, number of patients in the DC and no DC groups, the number of patients that developed PTH in each group, the DC type (unilateral or bilateral), the length of follow-up, and the definitions for PTH. Any discrepancies between the reviewers were resolved by a third investigator (B.A.).

### 2.4 Outcome assessed

We compared the incidence of development of PTH in patients who underwent DC versus those who received other treatment (non-DC group). We conducted two individual analyses; the first using data for all TBI patients, and the second using only data from studies with patients with severe (GCS<9) TBI. This was done to ensure inclusion of studies that included patients with all TBI severities.

It is important to note that, as a result of including observational studies, the patient groups (DC, non-DC) might present some baseline differences stemming from surgeons’ choice, patients’ characteristics, local and national health policies. It should also be noted that the main objective of some of the included studies was not to directly compare the DC versus non-DC groups.

### 2.5 Quality of the studies

We assessed the reporting quality and risk of bias using validated tools. All observational studies were assessed using the ROBINS tool (Sterne et al., 2016), while the Risk of Bias 2.0 (RoB 2.0) tool (Sterne et al., 2019) was used for RCTs. The overall quality of evidence was assessed according to the GRADE recommendations (Meader et al., 2014).

### 2.6 Statistical analysis

We used a paired meta-analysis to estimate the pooled odds ratio (OR) along with their 95% Confidence Interval (95% CI) to compare the incidence of PTH in the DC vs in the non-DC groups. Based on the presence of statistical heterogeneity, the meta-analysis was conducted according to fixed- or random effect models. In turn, the statistical heterogeneity of the studies was estimated by the use of the Cochran’s Q and *I*^2^ indices. When *I*^2^>50% and/or P_Q_ ​< ​0.10 the random effects model was used, otherwise the fixed effects model was implemented (Higgins and Thompson, 2002). We used funnel plots as well as the Egger’s and Begg’s tests to determine the existence of publication bias (Begg and Mazumdar, 1994; Egger et al., 1997; Mavridis and Salanti, 2014). The statistical significance was set at *p* ​< ​0.05. All statistical analyses were performed in Review Manager (Rev-Man) [Computer program], Version 5.3. Copenhagen: The Nordic Cochrane Centre, The Cochrane Collaboration, 2014.

## 3 Results

### 3.1 Selection of studies and study characteristics

**Fig. 1:**
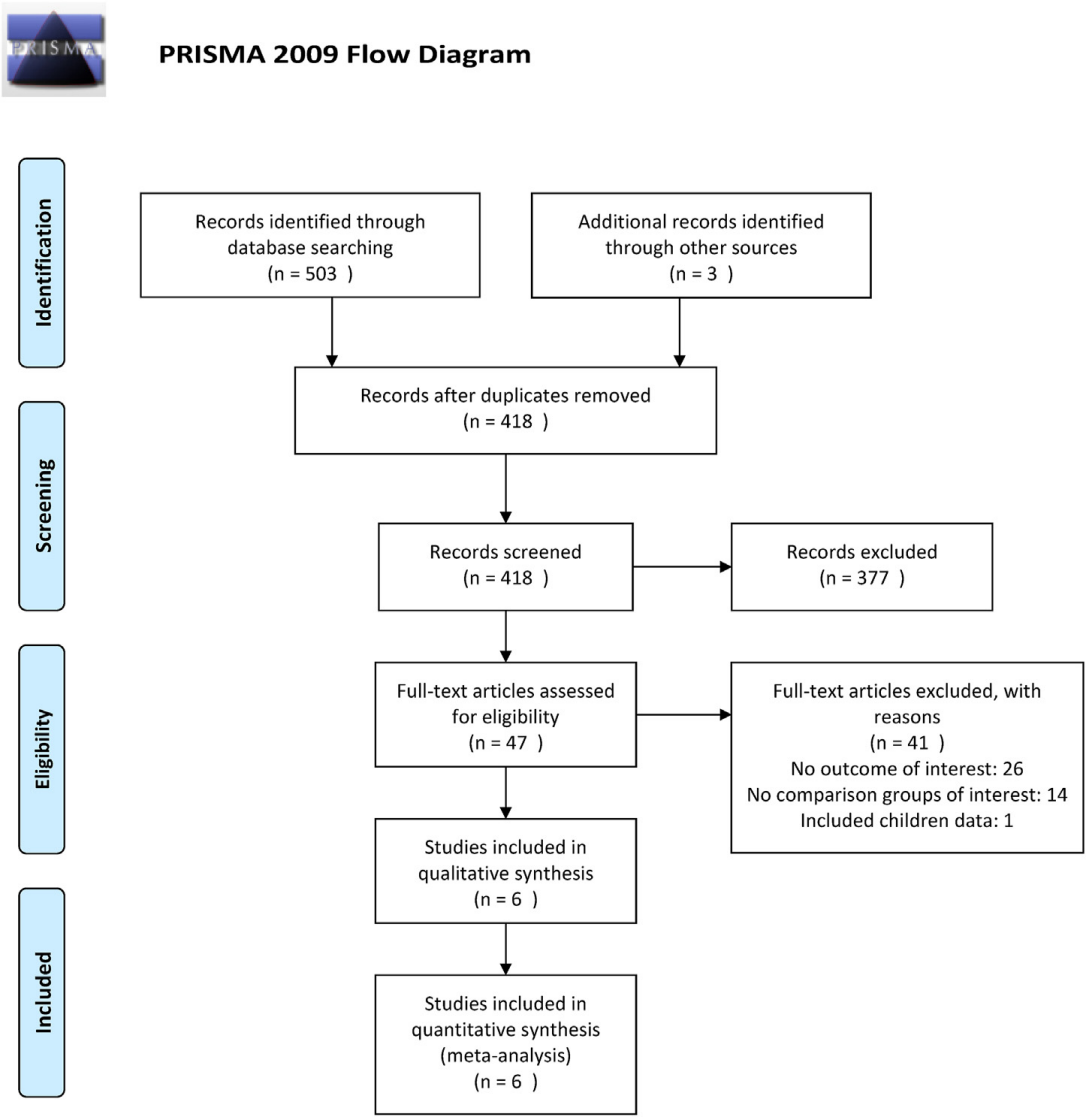
PRISMA flowchart presenting the study selection process.

Our literature search resulted in 418 individual articles, after the duplicates were removed. We excluded 377 and 41 studies after title and abstract screening and full-text reading, respectively. Finally, six articles (Chen et al., 2017; Choi et al., 2008; Cooper et al., 2011; Goldschmidt et al., 2020; Shi et al., 2011; Yuan et al., 2015) fulfilled our predetermined criteria and were included in our systematic reviews and meta-analysis (Fig. 1). A total of 2522 patients were included, 697 in the DC group and 1825 in the non-DC group. Out of the six studies included, five were observational and one was a RCT. Table 1 presents the main characteristics of the included studies.

**Table 1:** Table presenting the characteristics of the included studies.

First author - YOP	Country	Period of enrollment	Severity of TBI	Number of patients	Definition of hydrocephalus	Follow-up	Comments
Shi - 2011	China	2004–2010	severe	389	(Shunt surgery was performed based on) Dilation of ventricular system associated with periventricular lucency, but without enlargement of the cortical sulci and schizencephaly on dynamic CT examinations and progressive intracranial hypertension symptoms and signs, or manifestation of normal pressure hydrocephalous.	at least 6 months	–
Υuan - 2015	China	2009–2013	all levels of severity	379	Radiological evidence of progressive ventricular dilatation (Evans index >0.3) with trans-ependymal edema, together with the presence of either clinical deterioration or failure to make neurological progress over time and some evidence of clinical improvement after insertion of a ventriculo-peritoneal shunt.	at 3 months	–
Chen - 2017	China	2012–2015	all levels of severity	526	1) An Evans index (the largest width of the frontal horns of the lateral ventricles divided by the internal diameter of skull at the same level) greater than 0.3; 2) the enlargement of the anterior horns of the lateral ventricles, temporal horns and third ventricle, and periventricular interstitial edema in the presence of normal or absent sulci ​+ ​surgical flap tension in patients undergoing DC, neurobehavioral and cognitive disorders in conscious patients (e.g., inappropriate behavior, depressed mood, inability to plan or make a decision, memory or language disturbances) and no improvement or deterioration of consciousness in the comatose patients.	6 months	–
Goldschmidt - 2020	USA	2000–2014	severe	402	The need for a ventriculoperitoneal or subdural-peritoneal shunt.	12 months	–
Choi - 2008	Korea	2004–2007	all levels of severity	671	Radiographic evidence of ventricular dilatation on serial CT images in a patient whose clinical condition was deteriorating	Not defined	55 patients underwent DC, 33 included in the final analysis
Cooper - 2011	Australia, New Zealand, Saudi Arabia	2002–2010	severe	155	NA	6 months	–

Abbreviations. YOP: Year of Publication; TBI: Traumatic Brain Injury; CT: Computed Tomography; DC: Decompressive Craniectomy; NA: Not Available.

### 3.2 PTH development, all TBI patients

**Fig. 2:**
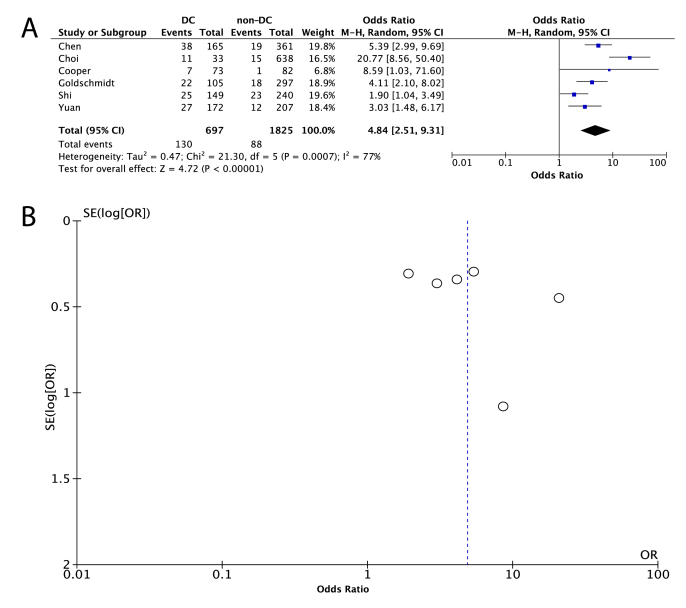
Presentation of the comparison of the incidence of post-traumatic hydrocephalus in the decompressive craniectomy (DC) group versus the group that was managed without DC (non-DC), when patients with all traumatic brain injury severities were taken into consideration. (A) Forest plot presenting the results of the analysis, (B) Funnel plot for the investigation of publication bias in this analysis.

Due to the presence of statistical heterogeneity (*I*^2^ ​= ​77%, P_Q_ ​= ​0.0007), the random effects model was used. Our analysis revealed that the odds of developing PTH were higher among patients undergoing DC than the odds of those in the non-DC group [OR (95% CI): 4.84 (2.51, 9.31)] (Fig. 2A). Based on funnel plots and statistical testing, no publication bias could be identified in this analysis (Fig. 2B, Table 2).

**Table 2:** Table presenting the results of the Egger’s and Begg’s tests for the investigation of publication bias.

Outcome	Egger’s test P value	Begg’s test P value
All severities of TBI	0.43	0.57
Severe TBI	0.57	0.6

### 3.3 PTH development, severe TBI patients

**Fig. 3:**
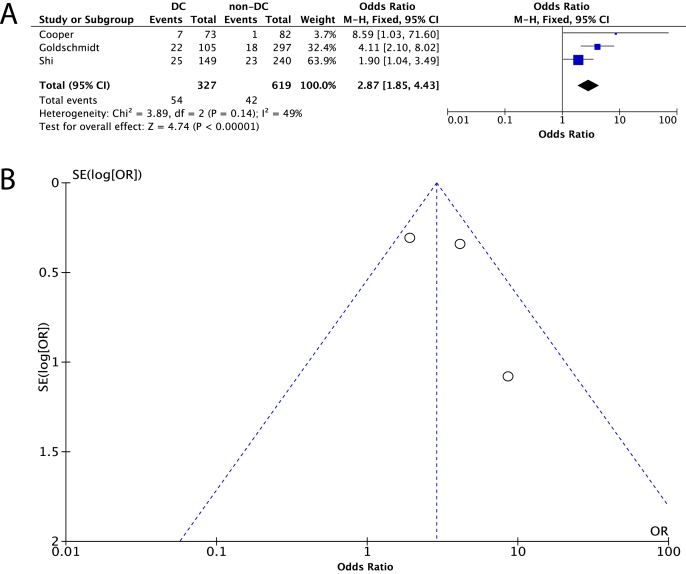
Presentation of the comparison of the incidence of post-traumatic hydrocephalus in the decompressive craniectomy (DC) group versus the group that was managed without DC (non-DC), when only patients with severe traumatic brain injury were taken into consideration. (A) Forest plot presenting the results of the analysis, (B) Funnel plot for the investigation of publication bias in this analysis.

In the absence of statistical heterogeneity (*I*^2^ ​= ​49%, P_Q_ ​= ​0.14), we used the fixed effects model in the re-analysis of our results when we focused solely on patients with severe TBI. The results showed that there was a higher probability of developing PTH among patients undergoing DC than in those who were treated without DC [OR (95% CI): 2.87 (1.85, 4.43)] (Fig. 3A). Based on funnel plots and statistical testing, no publication bias could be identified in this analysis, as well (Fig. 3B, Table 2).

### 3.4 Reporting quality of individual studies and overall quality

**Fig. 4:**
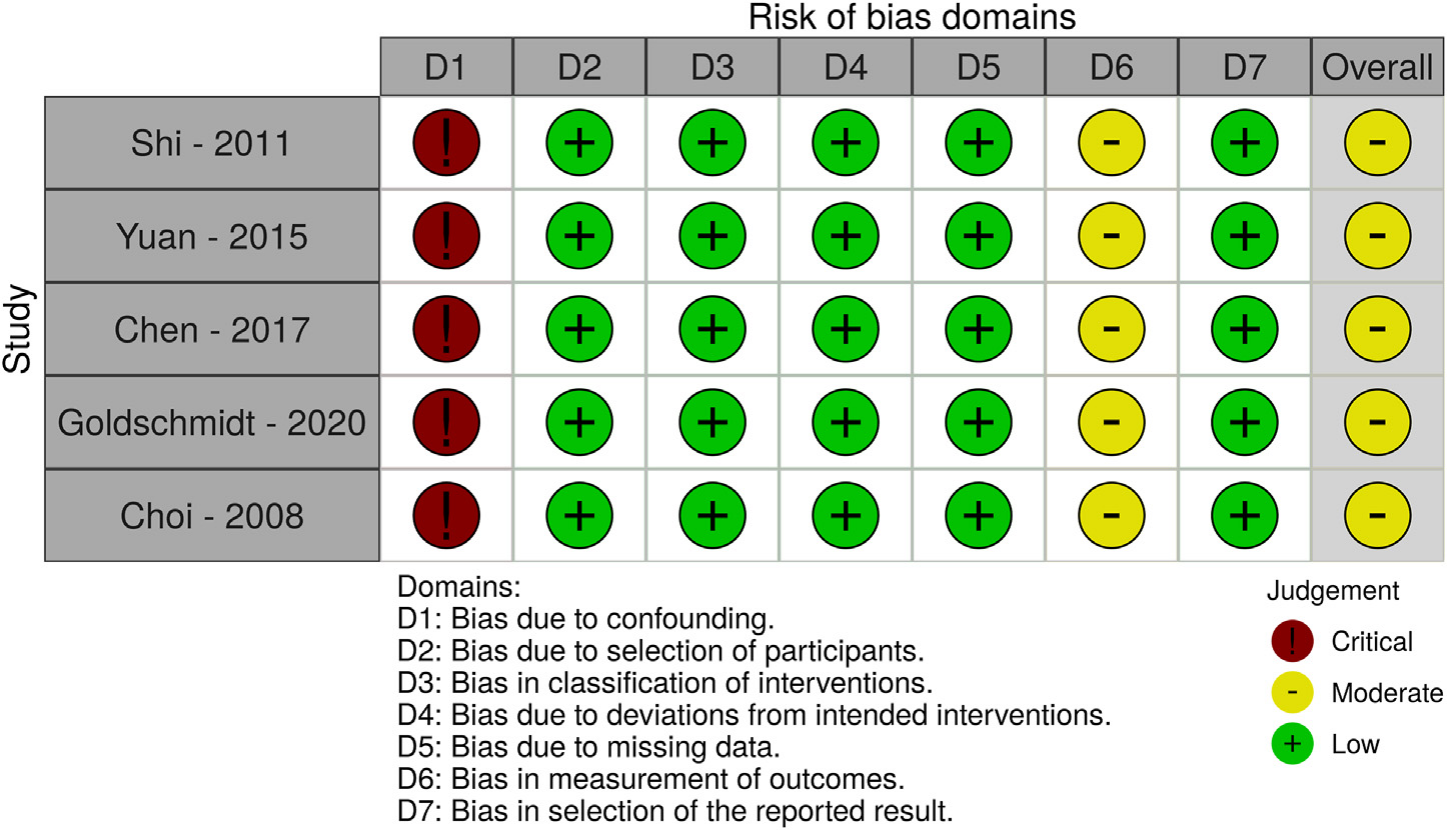
Graphical presentation of the results of the ROBINS assessment for observational studies.

**Fig. 5:**
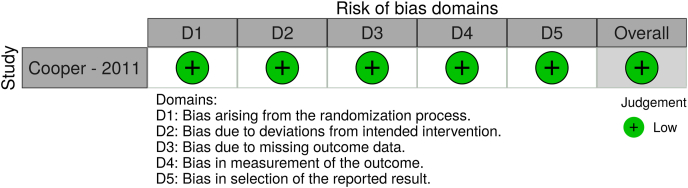
Graphical presentation of the results of the RoB 2.0 assessment for randomized controlled trials.

The reporting clarity and methodological quality of the gathered observational studies were characterized by a moderate level of bias for all the included studies, using the ROBINS tool. On the contrary, the RCT was associated with an overall low level of bias using the RoB 2.0 tool (Fig. 4, Fig. 5). Our results were based on moderate to low level overall quality of evidence, according to the GRADE recommendations (Table 3).

**Table 3:** Summary of the results of the systematic review and meta-analysis, alongside the outcome of the GRADE assessment.

Parameter under study	Number of studies	Number of patients	Pooled estimate – OR (95% CI)	GRADE quality of evidence	Comments
PTH (all TBI severities)	6	2522	4.84 (2.51, 9.31)	Moderate - Low	1. Further studies with a prospective design, predetermined strict inclusion and exclusion criteria, and a clearly defined hydrocephalus definition are needed.
PTH (severe TBI only)	3	946	2.87 (1.85, 4.43)	Moderate - Low	

## 4 Discussion

DC remains to this day as one of the few surgical treatment modalities available to neurosurgeons in order to manage refractory ICP that develops after TBI (Cooper et al., 2011; Hutchinson et al., 2016). However, although DC is effective in lowering ICP per se, it has been associated with more unfavorable outcomes, including the development of PTH, when compared with conservative/non-surgical management (Chen et al., 2017; Choi et al., 2008). Our systematic review and meta-analysis revealed that there is a higher probability of developing PTH among patients that underwent DC when compared to those that were managed without DC.

PTH as a result of TBI is a frequently observed phenomenon in neurosurgical and neurological clinical practice (Bonis and Anile, 2020; Poca et al., 2005). Two pathophysiological processes have been proposed as the main causes of ventricular enlargement after TBI; namely, brain atrophy secondary to diffuse axonal injury, and, abnormal CSF dynamics leading to true PTH (Guyot and Michael, 2000; Poca et al., 2005). Differentiating between brain atrophy and true PTH can often be challenging (Bonis and Anile, 2020). Various diagnostic modalities have been studied, such as ventricular or lumbar infusion tests, continuous ICP measurement, and single-photon emission computed tomography; however, none has become established in everyday clinical practice (Bonis and Anile, 2020; Gudeman et al., 1981; Mazzini et al., 2003; Osuka et al., 2010).

In a study by Lalou et al., the authors suggest that a classification of different forms of PTH can be made based on the time-phase after injury (Lalou et al., 2020). Acute PTH, that develops days to weeks after injury, can be the result of one of two processes: i) obstruction of normal CSF flow leading to ventricular enlargement and elevated ICP or ii) “external hydrocephalus”, due to CSF absorption impairment from the pacchionian granulations, leading to enlargement of cranial subarachnoid spaces with normal-sized ventricles (Manet et al., 2017). “Late phase PTH” is a result of the impairment of CSF circulation due to the inflammatory process that occurs after the traumatic event, and presents with ventricular enlargement and normal ICP (Czosnyka et al., 2000; Lalou et al., 2020).

In recent years, DC has been studied by some authors as a potential risk factor for the development of PTH (Ding et al., 2014). Several DC-related factors have previously been identified as potentially related to PTH development (Fotakopoulos et al., 2016), such as the distance of the medial edge of the craniectomy from the midline (De Bonis et al., 2010, 2013), the size of the craniectomy (Choi et al., 2008; Fotakopoulos et al., 2016), the presence of subdural hygroma (Honeybul and Ho, 2011), and delayed cranioplasty (Nasi et al., 2018). However, other authors couldn’t identify a causal effect between DC and PTH in their studies (Tian et al., 2008; Waziri et al., 2007). Our results confirm that patients that are managed with DC are more likely to develop PTH when compared with patients that are managed without DC.

The exact pathophysiological mechanism underlying the development of PTH after DC is incompletely understood. Waziri et al. (2007) reported that they observed a “flattening” of the normally dicrotic ICP waveform in patients after hemicraniectomy, possibly due to the transmission of the ICP pulse through the open cranial vault (Waziri et al., 2007). As a result, they speculated that this disruption of CSF dynamics can alter the one-way, pressure-dependent valve function of the arachnoid granulations (Upton and Weller, 1985), leading to diminished CSF outflow.

The present systematic review and meta-analysis has some limitations that should be acknowledged. The data included in the analysis were mostly extracted from observational studies rather than from RCTs, understandably resulting in a lower level of evidence. In fact, the absence of randomization introduces potential sources of selection bias. As a consequence, differences in the incidence of the PTH between the two groups might reflect differences in the treatment allocation and surgeon’s choice, based on the severity and qualitative characteristics of the underlying pathology. It is important to note that the included studies didn’t aim to compare the DC versus non-DC groups, they just reported the incidence of PTH in these groups. Consequently, they didn’t provide results of matching based on age, gender, presence of hematomas etc. Furthermore, as evident in Table 1, the definition of hydrocephalus varied between studies, while one study did not specify the definition used. The variety of definitions used could affect the overall calculated incidence of PTH. Studies that use definitions that only implement radiographic criteria (i.e., ventriculomegaly) for the diagnosis of PTH could identify a higher incidence. This could be misleading as, in some cases, the presence of ventriculomegaly does not have clinical significance; it does not reflect the clinical condition of the patient and does not manifest with clinical symptoms.

It should be noted that two of the included studies mainly studied adult patients, as evident by the reported mean/median age and standard deviation/interquartile range, but did not specify their exact age limit (Chen et al., 2017; Choi et al., 2008).

In conclusion, our results indicate that DC acts as a risk factor for the development of PTH. It is important that more studies with a prospective design should be conducted in order to provide high-level evidence on this topic.

## Funding

No funding was received for this research.

## Ethical standards

None applicable.

## Conflicts of interest

All authors certify that they have no affiliations with or involvement in any organization or entity with any financial interest (such as honoraria; educational grants; participation in speakers' bureaus; membership, employment, consultancies, stock ownership, or other equity interest; and expert testimony or patent-licensing arrangements), or non-financial interest (such as personal or professional relationships, affiliations, knowledge or beliefs) in the subject matter or materials discussed in this manuscript.

## Author contributions

Study conception and design: all authors. Material preparation, data collection and analysis: MG, KA, and BA. Drafting of the manuscript: MG and BA. All authors commented on previous versions of the manuscript. All authors read and approved the final manuscript. KF supervised the project.

## Declaration of competing interest

The authors declare that they have no known competing financial interests or personal relationships that could have appeared to influence the work reported in this paper.
