# Supplementary material for: Incidence of post-traumatic hydrocephalus in traumatic brain injury patients that underwent DC versus those that were managed without DC: A systematic review and meta-analysis
Source: Brain Spine. 2021 Oct 22;1:100303. doi: 10.1016/j.bas.2021.100303 (PMC9560681; doi:10.1016/j.bas.2021.100303)
Supplement: Multimedia component 2 [file mmc2.docx]

| **Database** | **PubMed** | **Scopus** | **clinicaltrials.gov** |
| --- | --- | --- | --- |
| **Exact algorithm used** | ("craniotomy"[MeSH Terms] OR "craniotomy"[All Fields] OR "craniectomies"[All Fields] OR "craniectomy"[All Fields] OR "decompressive craniectomy"[MeSH Terms]) AND ("craniocerebral trauma"[MeSH Terms] OR "brain injuries, traumatic"[MeSH Terms] OR "head injuries, closed"[MeSH Terms] OR "brain injuries"[MeSH Terms] OR "traumatic head"[All Fields] OR "traumatic brain"[All Fields] OR "head trauma"[All Fields] OR "brain trauma"[All Fields] OR "head injury"[All Fields]) AND ("hydrocephalus"[MeSH Terms] OR "post-traumatic hydrocephalus"[All Fields] OR "posttraumatic hydrocephalus"[All Fields]) | ( TITLE-ABS-KEY ( ( "craniectomies" OR "craniectomy" OR "decompressive craniectomy" ) ) ) AND ( TITLE-ABS-KEY ( ( "craniocerebral trauma" OR "traumatic brain injuries" OR "head injury" OR "brain injuries" OR "traumatic head" OR "traumatic brain" OR "head trauma" OR "brain trauma" ) ) ) AND ( TITLE-ABS-KEY ( ( "hydrocephalus" OR "post-traumatic hydrocephalus" OR "posttraumatic hydrocephalus" OR "post traumatic hydrocephalus" ) ) ) | ("hydrocephalus" OR "post-traumatic hydrocephalus" OR "posttraumatic hydrocephalus" OR "post traumatic hydrocephalus") AND "decompressive craniectomy" |
| **Number of results** | 174 | 328 | 1 |

Appendix B
